# Implications of ChatGPT in Public Health Dentistry: A Systematic Review

**DOI:** 10.7759/cureus.40367

**Published:** 2023-06-13

**Authors:** Anushree Tiwari, Amit Kumar, Shailesh Jain, Kanika S Dhull, Arunkumar Sajjanar, Rahul Puthenkandathil, Kapil Paiwal, Ramanpal Singh

**Affiliations:** 1 Clinical Quality and Value, American Academy of Orthopaedic Surgeons, Rosemont, USA; 2 Department of Dentistry, All India Institute of Medical Sciences, Patna, IND; 3 Department of Prosthodontics and Crown and Bridge, School of Dental Sciences, Sharda University, Greater Noida, IND; 4 Department of Pedodontics and Preventive Dentistry, Kalinga Institute of Dental Sciences (KIIT) Deemed to be University, Bhubaneswar, IND; 5 Department of Pediatrics and Preventive Dentistry, Swargiya Dadasaheb Kalmegh Smruti Dental College and Hospital, Nagpur, IND; 6 Department of Prosthodontics and Crown and Bridge, AB Shetty Memorial Institute of Dental Sciences (ABSMIDS) Nitte (Deemed to be University), Mangalore, IND; 7 Department of Oral and Maxillofacial Pathology, Daswani Dental College and Research Center, Kota, IND; 8 Oral Medicine and Radiology, New Horizon Dental College and Research Institute, Bilaspur, IND

**Keywords:** dentist, artificial intelligence, implications, public health dentistry, chatgpt

## Abstract

An artificial intelligence (AI) program called ChatGPT that generates text in response to typed commands has proven to be highly popular, as evidenced by the fact that OpenAI makes it available online. The goal of the present investigation was to investigate ChatGPT's potential applications as an outstanding instance of large language models (LLMs) in the fields of public dental health schooling, writing for academic use, research in public dental health, and clinical practice in public dental health based on the available data. Importantly, the goals of the current review included locating any drawbacks and issues that might be connected to using ChatGPT in the previously mentioned contexts in healthcare settings. Using search phrases including chatGPT, implications, artificial intelligence (AI), public health dentistry, public health, practice in public health dentistry, education in public health dentistry, academic writing in public health dentistry, etc., a thorough search was carried out on the Pubmed database, the Embase database, the Ovid database, the Global Health database, PsycINFO, and the Web of Science. The dates of publication were not restricted. Systematic searches were carried out for all publications according to inclusion and exclusion criteria between March 31, 2018, and March 31, 2023. Eighty-four papers were obtained through a literature search using search terms. Sixteen similar and duplicate papers were excluded and 68 distinct articles were initially selected. Thirty-three articles were excluded after reviewing abstracts and titles. Thirty-five papers were selected, for which full text was managed. Four extra papers were found manually from references. Thirty-nine articles with full texts were eligible for the study. Eighteen inadequate articles are excluded from the final 21 studies that were finally selected for systemic review. According to previously published studies, ChatGPT has demonstrated its effectiveness in helping scholars with the authoring of scientific research and dental studies. If the right structures are created, ChatGPT can offer suitable responses and more time to concentrate on the phase of experimentation for scientists. Risks include prejudice in the training data, undervaluing human skills, the possibility of fraud in science, as well as legal and reproducibility concerns. It was concluded that practice considering ChatGPT's potential significance, the research's uniqueness, and the premise-the activity of the human brain-remains. While there is no question about the superiority of incorporating ChatGPT into the practice of public health dentistry, it does not, in any way, take the place of a dentist since clinical practice involves more than just making diagnoses; it also involves relating to clinical findings and providing individualized patient care. Even though AI can be useful in a number of ways, a dentist must ultimately make the decision because dentistry is a field that involves several disciplines.

## Introduction and background

An artificial intelligence (AI) program called ChatGPT that constructs text in response to written instructions has been quite popular, as seen by its availability on the web via OpenAI (San Francisco, CA, USA) [1]. ChatGPT is a 175 billion-variable natural language recognition framework that employs deep learning techniques to produce responses that resemble those of people. It can handle a variety of themes because it is a dynamic interactive agent, making it suitable for chatbots, customer care, and other purposes. Although it has drawn a lot of curiosity for its unique capabilities, like producing Shakespearean sonnets, its failure to resolve basic mathematical problems has been noticed as well [2]. Similar to InstructGPT (a procedural pre-trained transformer system), ChatGPT was programmed using reinforcement training and human input.

In the medical field, paradigms of language have been investigated as instruments for public health education and individualized patient engagement. These kinds of models have potential, but they have not been successful in evaluating clinical expertise. The latest version of models offered by ChatGPT could more effectively bring together clinical expertise and dialogic communication. Creative uses made possible by its distinctive storytelling interface include acting as an inpatient, an idea-generation application, or another learner for small-group education. However, in order for these initiatives to be effective, ChatGPT must evaluate medical understanding and logic at a level that is comparable to that of humans [2] so that users may trust its responses. In previous research, we sought to assess how well medical students could perceive and understand ChatGPT. According to the research, ChatGPT's ability to comprehend and evaluate queries on parasitology exams is still below that of medical learners [3]. According to the researchers, ChatGPT was capable of addressing any query pertaining to the presented situation [4].

The generative teaching Pre-trained Transformer 3, which is considered GPT-3 x, from which ChatGPT derives its conceptual framework, has been educated on a sizable amount of data. It will be simple to access for many users globally, especially patients, nursing learners, medical learners, and doctors, thanks to its integration with the Microsoft Bing engine for searching [4]. Program directors should become aware of these technological advances' general characteristics and consider how they may affect undergraduate training in medicine, including the development of papers like personal assertions [5]. The research conducted earlier assessed the ability of ChatGPTs to assist in clinical choice-making in radiology by identifying acceptable diagnostic procedures for medical manifestations of breast discomfort and breast cancer surveillance [6]. The authors claim that it has been demonstrated to be feasible to implement ChatGPT for radiologic interpretations and that doing so has the potential to improve the workflow in hospitals while encouraging accountable utilization of radiological services [6,7-11].

It is generally known that humans have an inbuilt aversion to change, and this phenomenon may be understood from the viewpoints of social psychology and evolutionary psychology [12]. As a result, it seems reasonable that the worries and discussions that erupted right away after ChatGPT's widespread dissemination and the considerable amount of interest that ChatGPT attracted crossed many academic boundaries. For instance, in education, the release of ChatGPT may signal the termination of essays as homework [13].

The dangers of misuse, including the propagation of false information, ethical concerns, and factual mistakes, should all be taken into account in writing for academia and healthcare practice [14-16]. Human intelligence (HI) is more adaptable than AI due to its natural evolutionary background, versatility, inventiveness, psychological intelligence, and capacity for comprehending intricate abstract ideas [2]. However, HI-AI collaboration can be advantageous if a precise and reliable AI output is guaranteed. The possible use of AI to improve diagnostic and clinical decisions has already been discussed [17,18], along with its prospective benefits in personalized healthcare, the development of drugs, and the examination of enormous datasets. A fascinating area for research is the use of AI conversations in healthcare education. This corresponds to the vast amount of knowledge and different ideas that students studying health care must master [19].

All of these programs, however, should be carefully assessed in light of the legitimate worries, dangers, and paradigmatic failures encountered and mentioned in the larger context of large language model (LLM) submissions. Previous research carefully outlined the risks associated with using ChatGPT, which consisted of, but wasn't restricted to, the potential for biased and discriminatory behavior, the lack of accountability and dependability, cybersecurity issues, moral ramifications, and social repercussions [20]. The goal of the present investigation was to investigate ChatGPT's potential applications as an outstanding instance of LLMs in the fields of public dental health schooling, writing for academic use, research in public dental health, and clinical practice in public dental health based on the available data. Importantly, the goals of the current review included locating any drawbacks and issues that might be connected to using ChatGPT in the previously mentioned contexts in healthcare settings.

## Review

Search strategy

The Preferred Reporting Items for Systematic Reviews and Meta-Analyses (PRISMA) standards were followed in the preparation of the review. With the exception of an upgraded search method that increased the specificity of findings, there were no protocol violations. Using search phrases including chatGPT, implications, artificial intelligence (AI), public health dentistry, public health, practice in public health dentistry, education in public health dentistry, academic writing in public health dentistry, etc., a thorough search was carried out on the Pubmed database, Embase database, Ovid database, Global Health database, PsycINFO database, and Web of Science. The dates of publication were not restricted. Systematic searches were carried out for all publications according to inclusion and exclusion criteria between March 31, 2018, and March 31, 2023.

Reviewers were trained prior to being assigned for the screening of publications for eligibility in two steps (abstract-only screening and full-text analysis). Rayyan software was used to operate on an abstract-only inspection. Three reviewers (AB, CD, and EFG) each double-screened one-third of the search results, while one observer (XX) went through all of the results. Once abstracts were examined, reviewers got together to settle disagreements and come up with an ending list of articles to be read in full. Covidence software was used to perform a full-text review. The full-text articles were evaluated by CD and XX, two impartial raters, in relation to the requirements. When unsure whether an intervention used ChatGPT or AI, reviewers approached the researchers and looked for more details. Reviewers and committee directors of the study group reached an agreement on a final set of publications that would be considered (AB, CD).

Inclusion criteria and exclusion criteria

Articles, reviews, communications, editorials, and other types of preprints discussing ChatGPT that fit into one of the subsequent genres were eligible for inclusion: (1) practice and study in public dental healthcare; (2) education in public dental healthcare; and (3) writing for academic journals in public dental healthcare. The following were included as exclusion requirements: (1) documents not written in English; (2) documents on topics other than those listed in the inclusion criteria; and (3) publications from non-academic sources (such as websites, newspapers, and magazines).

Article selection

**Figure 1:**
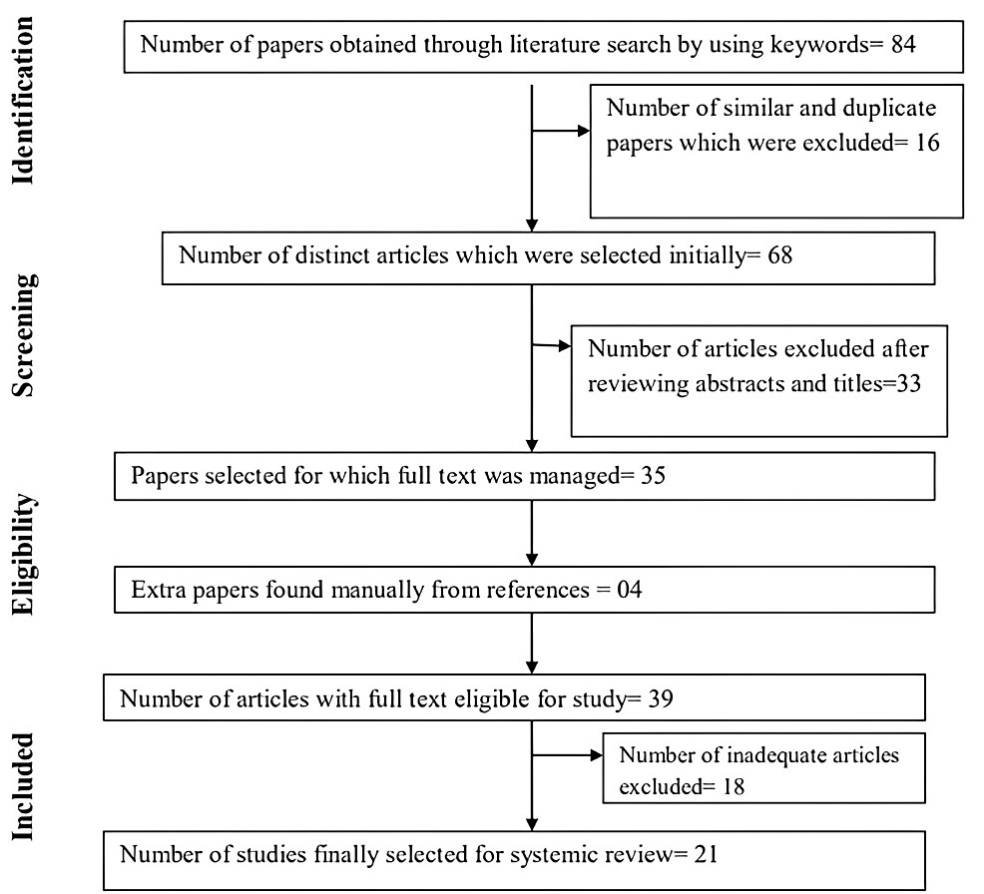
Preferred Reporting Items for Systematic Reviews and Meta-Analyses (PRISMA) flowchart showing selection of articles in this systematic review

Eighty-four papers were obtained through a literature search using search terms. Sixteen similar and duplicate papers were excluded. Sixty-eight distinct articles were initially selected. Thirty-three articles were excluded after reviewing abstracts and titles. Thirty-five papers were selected, for which full text was managed. Four extra papers were found manually from references. Thirty-nine articles with full texts were eligible for the study. Eighteen inadequate articles were excluded from the final. Twenty-one studies were finally selected for systemic review (Figure 1). Features of all articles included in the systematic review are shown in Table 1.

**Table 1:** Important features of all articles included in the systematic review

Authors	Aim, design	Observations, strengths, limitations	Conclusion
Jungwirth, D et al [1]	The goal of the research was to evaluate GPT-3's potential to improve public well-being and to determine whether deploying AI as a scholarly co-author was feasible.	We discovered that GPT-3 was capable of compiling, summarising, and producing convincing text blocks pertinent to issues of public health, illuminating useful domains of application for oneself. However, the majority of statements were wholly made up by GPT-3 and are therefore false. Our study shown that AI can be an important partner in research on public health. The AI was eventually not identified as a teamed up-author, as it was supposed to collaborate with another human researcher, in accordance with authorship requirements.	We come to the conclusion that proper scientific procedure must be followed when considering AI input, and that a robust scientific debate on AI accomplishments is required.
Giansanti D et al [2]	Intends to serve as a repository for academics to share information on challenges connected to the creation and application artificial intelligence in the field of public health including dental public health.	The significant proportion of reviews pertaining to these topics undoubtedly indicates that academics have an intense curiosity in social and scientific facilitation actions committed to the acceleration of study in this field with a view to both advancement and future prospective incorporation of AI. This is consoling and supports the notion of creating a Special Issue with this focus [8]. Both now and in the future, there are high expectations for AI, both in terms of its growth and its relationship with the health sector, which must also fulfil legal and ethical requirements as well as mainstream acceptance.	With the aid of AI, big statistics will enable us to recognise and address population habits as well as anticipate ailments on both individual and organisational levels. Wearable equipment will also enable us to monitor and gather personal medical data in order to adjust the procedure for providing care.
Fatani B [3]	To review all pertinent articles that have been written and discussed the usage of ChatGPT for healthcare and dentistry research .	The analysis of data and academic writing have begun to employ language models driven by AI [7]. ChatGPT, an AI language approach, may help medical academics and researchers with writing, literature study analysis, information summarization, organisation, citation, and title suggestions, as well as by creating an initial paper drafting.	According to previously published studies, ChatGPT has demonstrated its effectiveness in helping scholars with the authoring of scientific research and dental studies. Researchers have been able to summarise, interpret, and rephrase scientific data by using ChatGPT. However, it is not suggested to completely rely on ChatGPT for writing research papers as the scientific writing produced by the chatbot is still not thoroughly reviewed and additional research is needed to examine the ethical issues and unfavourable effects of this programme.
Sahrish Tariq [4]	To review the role of AI in public health dentistry	Digital dental assistants powered by artificial intelligence are capable of carrying out a number of duties more accurately, with fewer mistakes, and with less labour than people. Among these responsibilities are arranging and organising periodic visits to accommodate both patients and dentists, reminding patients as well as dentists to schedule checkups whenever genetic or lifestyle data point to increased vulnerability to dental problems, and helping with clinical evaluation and treatment planning. AI also promises to increase patient involvement in healthcare, particularly if they volunteer their data voluntarily. Self-management and monitoring one's own may encourage patients.	While there is no question about the superiority of incorporating AI into practise, it does not, in any way, take the place of a dentist since clinical practise involves more than just making diagnoses; it also involves relating to clinical findings and providing individualised patient care. Even though AI can be useful in a number of ways, a dentist must ultimately make the decision because dentistry is a field that involves several disciplines.
Islam NM [5]	Examines the possibilities as well as difficulties that dental education faces, putting a particular emphasis on integrating the incorporation of AI.	AI has a significant impact on education and healthcare, complementing and enhancing human labour. Adopting it could help existing operations and potential future innovation while also improving educational opportunities and healthcare delivery.	Even though the paradigm proposed in this research is particular to AI, it might be modified and utilised for a wide range of technologies as well as new organisational values and objectives inside dentistry education institutions.
Agrawal P et al [6]	To define AI in general terms and to discuss some potential applications for public health dentistry and dental education.	The use of AI is expanding to include many societal spheres.AI is used in speech detection, searches on the internet, and networking sites to customise the things you see and hear.AI is being used increasingly in the field of health care. AI is expanding into diagnostics and interpretation of images with more advanced technology. AI is being developed by researchers for application in all facets of dental care.	Dental clinics can become more productive by adopting AI to speed up tasks currently completed by the dental professional and assistant.
Thurzo, A et al [7]	A brief study of the significant improvements in the application of AI in dentistry education including public health dentistry since 2020, as well as a current summary of the impending changes	Naturally, the majority of educators in dentistry lack the understanding and skills necessary to evaluate applications based on AI because they weren't taught how to do so. Additionally, AI technology has advanced rapidly in the past few years. In the age of generative AI, factual accuracy and prospects with OpenAI Inc.'s ChatGPT are seen as crucial turning points. The clinical fields of dentistry will inevitably require updating as improved deep-learning algorithms transform diagnosis, treatment scheduling, leadership, and telemedicine surveillance.	Interaction with patients will alter as a result of recent developments in AI models of language, and the fundamentals of dental education, such as writing essays, theses, and scientific papers, will need to change as well. However, there is growing scepticism regarding its moral and legal ramifications, and more agreement is required for the secure and ethical adoption of artificial intelligence in the field of dentistry including public health dentistry.
Schwendicke F et al [8]	To develop an adequate number of goals that students should master when studying oral and dental artificial intelligence in order establish the fundamental syllabus for both undergraduates and post graduate education.	The learning outcomes fell into four categories, with the majority of them being "knowledge"-level results: (1) Basic concepts and definitions, (2) Training should cover use cases, the kinds of AI needed to solve them, and the normal configuration of artificial intelligence programmes for dentistry applications. (3) It is important to take into account evaluation measures, how they are understood, the relevant effects of artificial intelligence on patient or society health outcomes, and related cases. (4) It is important to emphasise issues with universality and representation, comprehensibility responsibility and independence, and the necessity for governance.	Planning, executing, and assessing oral and dental artificial intelligence (AI) instruction should take this basic curriculum into account for both instructors and students. .
Thurzo A et al, 2022 [9]	The first goal was to estimate how often artificial intelligence (AI), such as Chat Gpt, was used in dentistry literature between 2011 and 2021. The second goal was to identify the topic and field of dentistry of such publications, particularly dentistry.	The findings show that the growth in AI use in dentistry is increasing. All necessary conditions have been met, and enhanced neural networks may now be trained using digital workflows and digital big data. The use of artificial intelligence (AI) in dentistry is still viewed with scepticism by many clinicians; they believe it may be a hyped pattern with dubious credibility and little opportunity for beneficial contributions to the long-term advancement of the dental field.	The analysis indicates that artificial intelligence is currently mostly used in dentistry to evaluate digital diagnostic techniques, particularly radiology; nevertheless, its use is anticipated to increasingly permeate all areas of the profession.
Kumar [10]	A brief study and evaluation of ChatGPT for educational writing	Replies that are unique, methodical, and accurate; beneficial for training and for enhancing topic clarity; time-effective; and encouraging writers' motivation occasionally not following the directions correctly; Lack of in-text citations, incorrect citations, inadequate use of real-world examples, omission of highlights from one's own experiences, and cursory responses	Enhancing academic writing abilities, supporting universal concepts for learning, and using ChatGPT properly while receiving academic guidance are all possible benefits of ChatGPT.
Zielinski et al. [11]	Recommendations from WAME 1 for ChatGPT	For researchers, ChatGPT might serve as a useful tool.Risk of providing inaccurate or illogical replies; knowledge limited to the years prior to 2021; absence of legal individuality; the possibility of plagiarism	Authors must be open about their usage of ChatGPT and accept the liability of its content as ChatGPT does not adhere to ICMJE 4 standards. When editing ChatGPT-generated written material, editors require the proper detecting tools.
Biswas [12]	A future-looking account of healthcare literature in the context of ChatGPT	More efficiency when composing medical documents in context of public dental health. Inadequate knowledge of the medical area, ethical challenges, prejudice danger, legal challenges, and accountability problems	Despite being a potent instrument in the medical profession, ChatGPT has a number of drawbacks that should be taken into account.
StokelWalker [13]	An story about ChatGPT's perspective as an author.	The absence of responsibility, the possibility of plagiarism, and worries about abuse in academia	You shouldn't think of ChatGPT being an author of content.
Lund and Wang [14]	Report on the effects of ChatGPT in higher education	Helpful for conducting literature reviews, useful when performing data analysis, and helpful for translating ethical questions, data safety and confidentiality issues, discrimination risk, and accountability problems	Understand how to utilise ChatGPT sensibly and morally if you want to use it to progress academia.
Manohar and Prasad [15]	A case analysis created with help from ChatGPT	A crisp, understandable text was created with the aid of ChatGPT. Lack of scientific dependability and correctness; incorrect citations	Utilisation of ChatGPT is advised to avoid due to the possibility of inaccurate details and fictitious citations; it may be deceptive in medical practise.
Akhter and Cooper [16]	A case analysis created with help from ChatGPT	A pertinent broad introduction summary was made available with the aid of ChatGPT. Lack of access to pertinent literature, limited understanding up to 2021, inaccurate citations, and limited capacity for critical discussion of results	At this time, ChatGPT cannot take the place of impartial reviews of the literature in research in science.
Mann [17]	A viewpoint on role of ChatGPT in a translational study	Writing effectiveness, analysis of huge datasets (such as genetic data or digital medical records), prediction of illness risk factors, and disease outcomes prediction lowered data quality available; incapacity to comprehend how complicated biological systems are	In the foreseeable future, ChatGPT adoption in academic and medical and dental journals will be unavoidable.
Wang et al. [18]	An arXiv preprint 1 examines ChatGPT's ability to produce Boolean queries for comprehensive literature studies.	More accuracy compared to the approaches used for autonomous query generation currently A black-box utilisation, numerous inaccurate MeSH 11 terms, a lack of fit for high-recall access, and a wide range in query efficacy across different requests	A potential research instrument
Rao et al. [19]	A medRxiv publication on ChatGPT's utility in radiologic judgement	When determining the right imaging steps for breast cancer detection and assessment of breast pain, ChatGPT demonstrated fair accuracy. Insufficient references, consistency with user intent, misleading information, excessive detail, proposing imaging in pointless circumstances, providing justification for poor imaging judgements, and little transparency are all issues with this information.	It is possible to employ ChatGPT for radiographic decision-making, which could enhance the clinical procedure and promote ethical usage of radiology resources.
Sanmarchi et al. [20]	A preliminary paper on assessing the usefulness of ChatGPT in an epidemiological study that adheres to STROBE 9 recommendations	If the right structures are created, ChatGPT can offer suitable responses; greater time to concentrate on the phase of experimentation for scientists Risks include prejudice in the training data, undervaluing of human skill, the possibility of fraud in science, as well as legal and reproducibility concerns.	Considering ChatGPT's potential significance, the research's uniqueness and premise—the activity of the human brain—remain.
Lin [21]	A publication describing the usefulness of ChatGPT in academic settings	Variability Hallucination (false knowledge that appears convincing from a scientific perspective); unethical research; plagiarism danger; concerns about copyright.	Over the long run, ChatGPT has the potential to be revolutionary; accept it and make use of it to enhance human capacities, but what is most urgent are proper rules and norms of conduct.

Results

Characteristics of Manuscripts

The manuscripts included in this systematic review were original research, review articles, case reports and letters to editors. Some of the manuscripts discussed the role of ChatGPT in the practice of public health dentistry, while others discussed the role of ChatGPT in dental education in the field of public health dentistry. There were manuscripts that discussed other roles of ChatGPT in case studies, epidemiological studies, and translational research.

Chat GPT in Scientific Writing and Academia, Research, and Epidemiology in Public Health Dentistry

Kumar [10] presented a brief study and evaluation of ChatGPT for educational writing. They found replies that are unique, methodical, and accurate; beneficial for training and for enhancing topic clarity; time-effective; and encouraging writers' motivation; occasionally not following the directions correctly; lack of in-text citations; incorrect citations; inadequate use of real-world examples; omission of highlights from one's own experiences; and cursory responses. It was concluded that enhancing academic writing abilities, supporting universal concepts for learning, and using ChatGPT properly while receiving academic guidance are all possible benefits of ChatGPT.

Zielinski et al. [11] presented recommendations from World Association of Medical Editors 1 (WAME 1) for ChatGPT. They found that for researchers, ChatGPT might serve as a useful tool. Risk of providing inaccurate or illogical replies; knowledge limited to the years prior to 2021; absence of legal individuality; the possibility of plagiarism. They concluded that authors must be open about their usage of ChatGPT and accept the liability of its content, as ChatGPT does not adhere to International Committee of Medical Journal Editors 4 (ICMJE 4) standards. When editing ChatGPT-generated written material, editors require the proper detection tools. Stokel-Walker [13] presented a story about ChatGPT's perspective as an author. They found an absence of responsibility, the possibility of plagiarism, and worries about abuse in academia. It was concluded that one shouldn't think of ChatGPT as an author of content.

Manohar and Prasad [15] presented a case analysis created with help from ChatGPT. They found that a crisp, understandable text was created with the aid of ChatGPT. Due to lack of scientific dependability and correctness and incorrect citations, it was concluded that the use of ChatGPT is advised to be avoided due to the possibility of inaccurate details and fictitious citations; it may be deceptive in medical practice. Sanmarchi et al. [20] presented a preliminary paper on assessing the usefulness of ChatGPT in an epidemiological study that adheres to Strengthening the Reporting of Observational Studies in Epidemiology 9 (STROBE 9) recommendations. It was found that if the right structures are created, ChatGPT can offer suitable responses, giving scientists greater time to concentrate on the phase of experimentation. Risks include prejudice in the training data, undervaluing human skills, the possibility of fraud in science, as well as legal and reproducibility concerns. It was concluded that considering ChatGPT's potential significance, the research's uniqueness and premise-the activity of the human brain-remain.

Chat GPT in Dental Education in Public Health Dentistry

Thurzo et al. [9] conducted a study. The first goal was to estimate how often AI, such as ChatGPT, was used in dentistry literature between 2011 and 2021. The second goal was to identify the topic and field of dentistry in such publications, particularly dentistry. The findings show that the growth in AI use in dentistry is increasing. All necessary conditions have been met, and enhanced neural networks may now be trained using digital workflows and digital big data. The use of AI in dentistry is still viewed with skepticism by many clinicians; they believe it may be a hyped pattern with dubious credibility and little opportunity for beneficial contributions to the long-term advancement of the dental field. It was concluded that the analysis indicates that artificial intelligence is currently mostly used in dentistry to evaluate digital diagnostic techniques, particularly radiology; nevertheless, its use is anticipated to increasingly permeate all areas of the profession.

Thurzo et al. [7] conducted a brief study of the significant improvements in the application of AI (ChatGPT) in dentistry education, including public health dentistry, since 2020, as well as a current summary of the impending changes. It was found that, naturally, the majority of educators in dentistry lack the understanding and skills necessary to evaluate applications based on AI because they weren't taught how to do so. Additionally, AI technology has advanced rapidly in the past few years. In the age of generative AI, factual accuracy and prospects with OpenAI Inc.'s ChatGPT are seen as crucial turning points. The clinical fields of dentistry will inevitably require updating as improved deep-learning algorithms transform diagnosis, treatment scheduling, leadership, and telemedicine surveillance. It was concluded that interaction with patients will alter as a result of recent developments in AI models of language, and the fundamentals of dental education, such as writing essays, theses, and scientific papers, will need to change as well. However, there is growing skepticism regarding its moral and legal ramifications, and more agreement is required for the secure and ethical adoption of artificial intelligence in the field of dentistry, including public health dentistry.

Schwendicke et al. [8] conducted a study to develop an adequate number of goals that students should master when studying oral and dental artificial intelligence in order to establish the fundamental syllabus for both undergraduate and postgraduate education. The learning outcomes fell into four categories, with the majority of them being "knowledge"-level results: (1) Basic concepts and definitions, (2) Training should cover use cases, the kinds of AI needed to solve them, and the normal configuration of artificial intelligence programs for dentistry applications, (3) It is important to take into account evaluation measures, how they are understood, the relevant effects of artificial intelligence on patients' or society's health outcomes, and related cases, (4) It is important to emphasize issues with universality and representation, comprehension, responsibility, and independence, as well as the necessity for governance. Planning, executing, and assessing oral and dental AI instruction should take this basic curriculum into account for both instructors and students.

Agrawal et al. [6] presented a manuscript to define AI in general terms and to discuss some potential applications for public health dentistry and dental education. It was observed that the use of AI is expanding to include many societal spheres. AI is used in speech detection, searches on the internet, and networking sites to customize the things you see and hear. AI is being used increasingly in the field of health care. AI is expanding into diagnostics and the interpretation of images with more advanced technology. AI is being developed by researchers for application in all facets of dental care. It was concluded that dental clinics can become more productive by adopting AI to speed up tasks currently completed by dental professionals and assistants.

ChatGPT in the Practice of Public Health Dentistry

Fatani [3] conducted a study to review all pertinent articles that have been written and discuss the usage of ChatGPT for healthcare and dentistry research. It was found that the analysis of data and academic writing have begun to employ language models driven by AI. ChatGPT, an AI language approach, may help medical academics and researchers with writing, literature study analysis, information summarization, organization, citation, and title suggestions, as well as by creating an initial paper draught. According to previously published studies, ChatGPT has demonstrated its effectiveness in helping scholars with the authoring of scientific research and dental studies. Researchers have been able to summarise, interpret, and rephrase scientific data by using ChatGPT. However, it is not suggested to completely rely on ChatGPT for writing research papers as the scientific writing produced by the chatbot is still not thoroughly reviewed and additional research is needed to examine the ethical issues and unfavorable effects of this program.

Giansanti et al. [2] conducted a study that intends to serve as a repository for academics to share information on challenges connected to the creation and application of artificial intelligence in the field of public health, including dental public health. It was found that the significant proportion of reviews pertaining to these topics undoubtedly indicates that academics have an intense curiosity in social and scientific facilitation actions committed to the acceleration of study in this field with a view to both advancement and future prospective incorporation of AI. This is consoling and supports the notion of creating a special issue with this focus.

Both now and in the future, there are high expectations for AI, both in terms of its growth and its relationship with the health sector, which must also fulfill legal and ethical requirements as well as mainstream acceptance. Tariq [4] conducted a study to review the role of AI in public health dentistry. It was found that digital dental assistants powered by artificial intelligence are capable of carrying out a number of duties more accurately, with fewer mistakes, and with less labor than people. Among these responsibilities are arranging and organizing periodic visits to accommodate both patients and dentists, reminding patients as well as dentists to schedule checkups whenever genetic or lifestyle data point to increased vulnerability to dental problems, and helping with clinical evaluation and treatment planning. AI also promises to increase patient involvement in healthcare, particularly if they volunteer their data voluntarily. Self-management and monitoring one's own may encourage patients. It was concluded that while there is no question about the superiority of incorporating AI into practice, it does not, in any way, take the place of a dentist since clinical practice involves more than just making diagnoses; it also involves relating to clinical findings and providing individualized patient care. Even though AI can be useful in a number of ways, a dentist must ultimately make the decision because dentistry is a field that involves several disciplines.

Discussion

The goal of the present investigation was to investigate ChatGPT's potential applications as an outstanding instance of LLMs in the fields of health schooling, writing for academic use, research in healthcare, and clinical practice based on the available data. Importantly, the goals of the current review included locating any drawbacks and issues that might be connected to using ChatGPT in the previously mentioned contexts in healthcare settings. Some of the manuscripts discussed the role of ChatGPT in the practice of public health dentistry, while others discussed the role of ChatGPT in dental education in the field of public health dentistry. There were manuscripts that discussed other roles of ChatGPT in case studies, epidemiological studies, and translational research.

There were some excellent outcomes regarding the use of ChatGPT in academia and scientific writing; however, some drawbacks were also noticed, and many studies concluded that it would be too early to consider ChatGPT as an author. A brief analysis and evaluation of ChatGPT for educational writing were offered by Kumar [10]. They discovered responses that are original, methodical, and accurate; helpful for training and for enhancing topic clarity; time-effective; and encouraging writers' motivation by occasionally not following the directions correctly. They also discovered responses that are brief and devoid of in-text citations, incorrect citations, insufficient use of real-world examples, inadequate use of highlights from one's own experiences, and cursory responses. It was determined that ChatGPT might improve academic writing skills, enhance universal learning principles, and be used correctly while obtaining academic supervision. In contrast, Zielinski et al. [11] reported ChatGPT suggestions from WAME 1. They discovered that ChatGPT can be a helpful tool for researchers. Risk of responding with incorrect or illogical information; knowledge restricted to the years up to 2021; lack of legal distinctiveness; potential for copying. They came to the conclusion that, as ChatGPT does not comply with international committee of medical journal editors (ICMJE 4) requirements, authors must be transparent about their use of it and accept responsibility for its content. Editors need the right detection tools to modify textual content produced by ChatGPT. Similar to this, Stokel-Walker [13] told a tale from ChatGPT's point of view as an author. They discovered a lack of accountability, the potential for plagiarism, and concerns about abuse in academia. It was concluded that one shouldn't think of ChatGPT as an author of content. The role of ChatGPT in dental education and public health education was also discussed, with varying results. Some studies showed beneficial effects along with drawbacks. The research was done by Thurzo et al. The initial objective was to gauge the frequency with which AI, such as ChatGPT, was mentioned in dental literature from 2011 to 2021. The second objective was to determine the subject and area of dentistry, specifically dentistry, of such publications. The results indicate that the application of AI in dentistry is expanding. Now that all prerequisites have been satisfied, improved neural networks can be trained with the use of digital workflows and large amounts of data. Many dentists are still skeptical of the use of AI in dentistry; they think it might be a hyped trend with questionable legitimacy and a limited chance of making positive contributions to the long-term growth of the dental sector. The investigation found that artificial intelligence is presently mostly employed in dentistry to assess digital diagnostic techniques, particularly in radiology; however, its usage is projected to spread more widely throughout the profession as time goes on. Similar to this, Schwendicke et al. [8] carried out a study to define the essential curriculum for both undergraduate and postgraduate education and to develop an acceptable number of goals that students should master when studying oral and dental artificial intelligence. The learning outcomes were divided into four groups, the bulk of which were "knowledge"-level outcomes: (2) Training should cover use cases, the types of AI needed to answer them, and the typical configuration of artificial intelligence programs for dental applications, in addition to basic ideas and terminology, (3) It's crucial to consider evaluation criteria, how they're interpreted, the pertinent impacts of artificial intelligence on patient or societal health outcomes, as well as relevant situations, (4) It's critical to highlight concerns about universality and representation, understandability, accountability and independence, and the need for governance. This fundamental curriculum should be taken into consideration while planning, carrying out, and evaluating oral and AI teaching for both instructors and students.

When there was an analysis of the role of ChatGPT, an artificial intelligence-driven application, it was observed that although this application offers some great advantages, it also has certain limitations. A study was carried out to examine the function of AI in public health dentistry by Tariq [4]. It was discovered that digital dental assistants equipped with artificial intelligence are capable of doing a variety of tasks more efficiently, precisely, and with fewer errors than people. These duties include scheduling routine appointments for both patients and dentists, reminding both parties to schedule checkups whenever genetic or lifestyle information indicates a higher risk of dental problems, and assisting with clinical evaluation and treatment planning. Additionally, AI holds out the prospect of increasing patient involvement in healthcare, especially if they actively provide their data. Patients may be encouraged by practicing self-management and self-monitoring.

The researchers came to the conclusion that while integrating AI into practice is undoubtedly superior, a dentist is still necessary because clinical practice entails more than just making diagnoses; it also entails relating to clinical findings and offering individualized patient care. A dentist must finally make the decision because dentistry is a specialty that incorporates numerous disciplines, even if AI can be helpful in a number of ways. The flaws with this systematic review are: (1) the quality of the included records can vary, which limits the results' generalizability; (2) the removal of records in languages other than English may have caused selection prejudice; and (3) the marginalization of a few records that were not accessible may have left out important information despite their rarity.

## Conclusions

According to previously published studies, ChatGPT has demonstrated its effectiveness in helping scholars with the authoring of scientific research and dental studies. Researchers have been able to summarise, interpret, and rephrase scientific data by using ChatGPT. However, it is not suggested to completely rely on ChatGPT for writing research papers as the scientific writing produced by the chatbot is still not thoroughly reviewed and additional research is needed to examine the ethical issues and unfavorable effects of this program. Over the long run, ChatGPT has the potential to be revolutionary; accept it and make use of it to enhance human capacities, but what is most urgent are proper rules and norms of conduct. If the right structures are created, ChatGPT can offer suitable responses, giving scientists greater time to concentrate on the phase of experimentation. Risks include prejudice in the training data, undervaluing human skills, the possibility of fraud in science, as well as legal and reproducibility concerns. It was concluded that considering ChatGPT's potential significance, the research's uniqueness and premise-the activity of the human brain-remain. While there is no question about the superiority of incorporating ChatGPT into the practice of public health dentistry, it does not, in any way, take the place of a dentist since clinical practice involves more than just making diagnoses; it also involves relating to clinical findings and providing individualized patient care. Even though AI can be useful in a number of ways, a dentist must ultimately make the decision because dentistry is a field that involves several disciplines.
